# Lumbo-Pelvic Dissociation in a Patient With Complex Spinal Deformity: A Case Report

**DOI:** 10.7759/cureus.30547

**Published:** 2022-10-21

**Authors:** Robert T Rella, Jesse Trent, Richard Menger

**Affiliations:** 1 College of Medicine, University of South Alabama College of Medicine, Mobile, USA; 2 Orthopedic Surgery, University of South Alabama College of Medicine, Mobile, USA; 3 Neurological Surgery, University of South Alabama College of Medicine, Mobile, USA

**Keywords:** spinal deformity, revision, spinal trauma, hardware malufunction, hardware infection, lumbar spine surgery, spondyloptosis, complex spine deformities, pelvic anteversion, lumbopelvic dissociation

## Abstract

Lumbopelvic dissociation is an extremely rare injury to the junction of the lumbar spine and sacrum seen in high-energy trauma, for which the operative treatment has not been established, especially in the setting of hardware infection. In this case report, we describe the case of a 37-year-old male who presented to the spine surgery team after undergoing six surgeries, all following a traumatic car accident ten years prior.

The patient initially presented with symptomatic lumbar hyperlordosis that had progressively limited his ability to perform activities of daily living. He suffered from paraplegia and a sensory deficit at the T8 level and below but still maintained control over his bowel and bladder. The surgical team performed two operations: one to improve his quality of life by correcting the degree of lordosis he was suffering from due to a 76-degree sacral slope and the second to perform re-instrumentation after the patient suffered a traumatic injury three weeks after the initial operation that occurred after assisting with his own wheelchair transfers.

His prior surgeries include operations for deformity correction as well as irrigation and debridement secondary to hardware infection and subsequent removal. He reported that following the hardware removal he had significant pain and was no longer able to easily sit and play with his child or reach countertops while in his wheelchair, severely impacting his quality of life.

The surgical team performed two operations on this patient: the first to correct the lordotic deformity utilizing a four-rod construct, and a second performed three weeks later to perform re-instrumentation utilizing a five-rod construct and hematoma evacuation following hardware failure secondary to high biomechanical strain from performing his own wheelchair transfers.

## Introduction

Lumbopelvic dissociation is a rare spinal deformity associated with high-energy traumas, such as motor vehicle accidents, combat injuries, or falls from height. The injury has a high association with many other injuries including neurological deficits producing a multisystem impact. Both conservative and operative interventions have been described without consensus on the standard of care [1,2-6].

While there is no established treatment algorithm for these injuries [4], efforts to classify these injuries have been made to facilitate clinical decision-making [7,8]. The operative intervention has been associated with earlier mobilization and better mechanical stability acutely. However, there is a relatively high infection rate as could be expected due to the soft tissue damage [2-4,8,9,10-12]. There is at least one case treated conservatively with late fixation reporting a favorable clinical outcome [1].

While lumbopelvic dissociation injuries and treatments are well documented, the removal of infected hardware after correction of lumbopelvic dissociation is not. We present a 37-year-old male with a severe sacral slope in the setting of numerous surgeries for lumbopelvic dissociation, including the removal of hardware secondary to infection and correction of hardware failure following high strain postoperatively.

The initial operation included a posterior approach to perform fixation from T8 to the pelvis as well as the removal of prior posterior spinal instrumentation. Using a cantilever technique, significant traction was applied in order to retrovert the pelvis and correct the lordotic deformity. Postoperative X-rays displayed a resultant sacral slope of approximately 51 degrees.

At the two-week follow-up, the patient reported feeling remarkably well and he was able to play with his child again as well as reach objects on the countertops and sink that were previously unreachable. Despite adamant advisement against excessive activity, the patient returned to the emergency department (ED) after feeling a large "popping" sensation three weeks postoperatively. Due to the lack of social support, the patient was forced to go against our medical advice and perform independent wheelchair transfers. The high biomechanical strain placed on the four-rod construct caused his pelvis to fracture along with a frank rod dislodgement.

The patient was urgently taken to the operating room (OR) for a second time to perform re-instrumentation and evacuate the subsequent hematoma. The pelvis was re-instrumented with six points of pelvic fixation, an increase from four during the first operation. Another rod was added to create a five-rod complex supporting his spine and pelvis. The patient was advised to remain on strict bedrest for the next six weeks during fusion healing. The patient has not reported any major adverse events one year after the revision procedure.

## Case presentation

Initial patient presentation

A 37-year-old male was in a motor vehicle collision approximately 10 years ago that resulted in a T8 American Spinal Injury Association (ASIA) B injury. He had undergone six previous spine surgeries for initial deformity correction with a large posterior decompression and fusion. The other operations included secondary irrigation and debridement and eventual partial hardware removal secondary to osteomyelitis of the lumbar spine. He was on oral maintenance antibiotics indefinitely. He presented with worsening lumbosacral back pain that prevented him from sitting in his wheelchair for longer than two hours at a time and prevented him from playing with his son. He also reported being unable to sit up straight as well as multiple falls due to his feet lodging underneath his wheelchair. On physical exam patient was alert and oriented x3, strength was 5/5 bilaterally in the upper extremities, 0/5 bilaterally in the lower extremities with an ASIA B defect with impaired motor and partial sensation deficit below dermatome T8 but was continent of bowel and bladder. He had a prominent amount of scar tissue built up on his back due to the number of previous surgeries.

**Figure 1:**
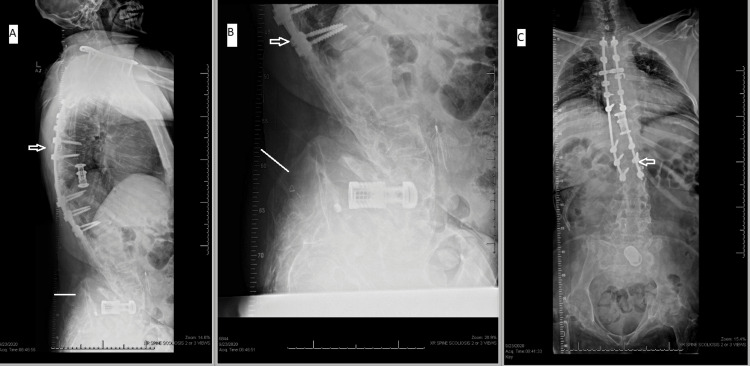
Preoperative radiographs and prior instrumentation A. Lateral view of the entire spine including previously installed hardware (arrow) and the superior region of the pelvis (line). B. Lateral view of the lumbar spine with instrumentation (arrow) and the pelvis (line). C. Anteroposterior radiograph of the entire spine and pelvis demonstrating prior instrumentation (arrow).

Anteroposterior and lateral radiographs showed a hook-rod hybrid construct in the thoracic spine. There appeared to be a completely rotated lumbar cage with retropulsion into the spinal canal. This came on a pelvis that had a near 76-degree sacral slope, a positive sagittal imbalance of approximately 4.3 cm (Figure 1). Over time, the patient’s pelvis tilt had drifted anteriorly according to prior imaging. This demonstrated significant shear stress on the area, which has likely been the source of his pain.

Operation 1

All neuromonitoring measures were at baseline for this patient’s operation. A central incision was made from T8 to the sacrum at S2 with subperiosteal dissection down to the lateral border of the pars interarticularis and medial border of the transverse processes. The diffusion mass was encountered and discarded. His prior instrumentation was stable with an associated proximal fusion mass. His pelvis was anteverted, consistent with prior imaging. A dissection was performed without contacting frank pus. The corpectomy cage at L5 was visualized and an X-ray was taken to confirm positioning. Based on the location, the X-ray showed a correction of approximately 20% in deformity. Then, we placed our attention on replacing the lumbar screws which proved to be challenging as the landmarks were completely obliterated by fusion mass. We used fluoroscopic guidance to assist in finding trajectories which would allow us to safely place the largest screws possible. The screws were placed in a lateral to medial trajectory to improve the overall pullout strength. No neuromonitoring changes were observed when we placed the L1-L3 pedicle screws, which were found to have excellent purchase.

Next, attention was turned to pelvic instrumentation. The anteverted pelvis was exposed laterally and posteriorly. It was noted that the S1 lamina was approaching a right angle. We placed four pelvis screws (two bilaterally) using a teardrop Judet view to ensure the correct trajectory. The pelvis screws were found to have a good purchase as the pelvis was manipulated.

Following was an inspection of the corpectomy cage. Despite the cage being somewhat loose posteriorly, we were still unable to easily remove the cage. There was no disruption to the thecal sac or cerebrospinal fluid (CSF) leak. Consequently, we decided to proceed with a wide decompression of the cage to facilitate its removal. Finally, we proceeded to break out the fusion mass at L5-S1 laterally.

Given the patient’s coronal deformity, a working rod was placed on the right side and then locked the lumbar screws utilizing set screws. We then used the fusion mass to cantilever his pelvis. Using distraction, we took the patient’s pelvis out of frank anteversion, and the pelvis moved freely without issue. No change in hemodynamic status or neuromonitoring was encountered during these maneuvers. After having appropriate distraction on the right, the process was repeated on the left with a working rod placed and eventually even positioning was achieved. We distracted the pelvis back and forth. Once seated, we then advanced to place our other rods in the quad-rod technique. We proceeded to domino to T8, being sure to cross-link on the right side to ensure the biomechanical load was shared. Next, we decorticated the fusion mass and lumbar area. We used a donor fibular graft on the defect as well as a significant amount of I factor secondary to the high pseudoarthrosis rate. Finally, the patient was given Cell Saver blood back. Plastic surgery then closed the wound in layers to decrease the risk of infection.

Postoperative course 1

The patient was taken to the intensive care unit (ICU) in a guarded but stable fashion, where he was observed until postoperative day (POD) 3 when he was then transferred to the floor. He remained stable throughout his stay, however, he was diagnosed with a urinary tract infection (UTI) on POD 4 and was started on meropenem. He received packed red blood cells on POD 6 secondary to blood loss anemia. He had relatively high drain outputs on POD 4-7. He was eventually discharged home without further incident on POD 9.

He was seen in the clinic on POD 17 and reported doing remarkably well. He reported better pain control, being able to sit up straight as well as being able to reach objects on counters that he had not previously been able to reach. Most importantly, he reported that he was able to tolerate sitting longer, which allowed him to play with his son for longer periods of time. He was informed that his construct was under high strain and was instructed to refrain from assisting in transfers at all. He reported that he was doing his best at that time but due to his socioeconomic situation he was still performing some independent transfers.

**Figure 2:**
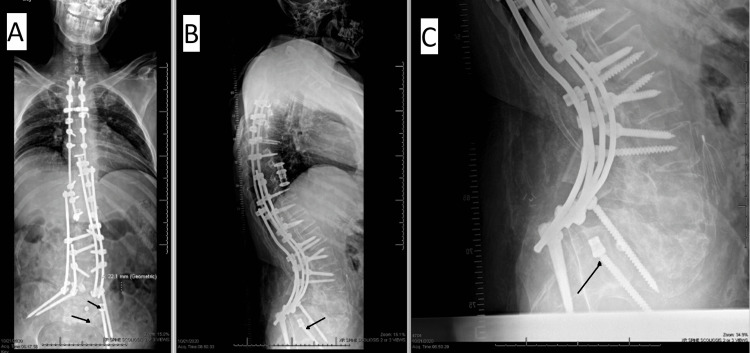
Radiographs demonstrating the lumbopelvic dissociation injury 26 days after the initial operation performed. A: Anteroposterior radiograph of the entire spine and construct. The short, superior arrow denotes the dislodged pelvic screw, and the long, inferior arrow denotes the inferior margin of the fracture line. The superior region of the pelvic fracture line can also be seen with the white dotted line. B: Lateral radiograph of the entire spine with the arrow denoting the pelvic screw that dislodged from the rod construct. C: Lateral radiograph of the lumbopelvic dissociation with the arrow again denoting the dislodged pelvic screw.

On POD 26 patient returned to the clinic and reported a significant “popping” event while transferring from his wheelchair independently. Radiographs revealed a fracture in his pelvis as well as dissociation of one of the iliac screws from the rod on the left side of the construct (Figure 2). He maintained good alignment and with three points of fixation in the pelvis. It was determined that obtaining a bone stimulator might be the best plan moving forwards.

On POD 29, the patient was readmitted to the hospital secondary to a large hematoma formation over his lower back. The spine surgery team planned a hardware revision while the plastic surgery team planned irrigation and debridement.

Operation 2

A large, raised fluctuant mass was encountered over the patient's lower back and pelvis. The plastic surgery team performed the incision and drainage of the hematoma. Afterwards, the spine surgery team turned their attention to the damaged posterior instrumentation. This damage included both iliac fixation points on the left as well as the medial fixation on the right. The pelvis was visibly fractured through the left iliac crest into the pelvic inlet.

We used navigation through the iliac crest to aid us in our pelvic instrumentations. Every screw was placed to facilitate a rod construct closure. On the right side, the two previous iliac screws were found to be in a good position with good purchase, so one more iliac screw was placed medial to the previous two screws. On the left side, the lateral iliac screw was left in place while two more iliac screws were placed medially to it after the failed hardware was removed. This allowed us to create and then domino a five-rod construct in multiple planes. We specifically connected the different rod structures to facilitate load sharing. The weakest rod in our construct was on the far left. Therefore, we used cross-connectors to share the biomechanical load.

**Figure 3:**
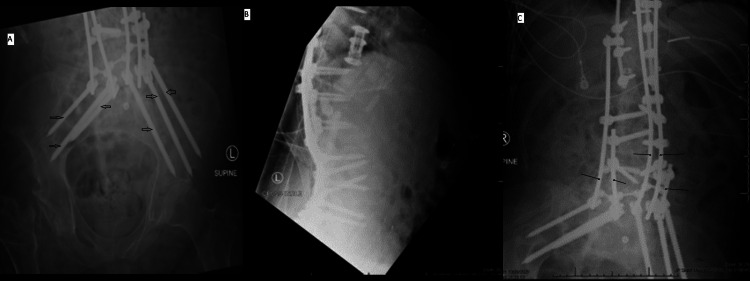
Postoperative radiographs following the second operation The pelvic fracture and lumbopelvic dissociation were corrected by adding another rod to create a five-rod construct and increasing the number of pelvic fixation points to six, up from four previously. A. Anteroposterior radiograph of the pelvis with each point of fixation denoted (arrows). B. Lateral radiograph of the pelvis. C. Anteroposterior radiograph of the lumbar spine and superior pelvis, with all five rods in the construct denoted (lines).

After we tightened all screws and covered all rod constructs, it was determined that this construct was much more stable than the first. No deformity correction was performed in the second operation as it felt to be unsafe after the hardware failure following the first operation. The patient was then taken to the ICU in stable condition and postoperative radiographs are included (Figure 3).

Postoperative course 2

Postoperatively, the patient did well without concern. He was discharged to home on POD 6 with home health services and orders for six weeks of strict bed rest. The patient has yet to present with any traumatic issues in over a year of follow-up, likely due to the increased stability of the five-rod construct and better help at home transferring in and out of his wheelchair.

## Discussion

Traumatic lumbopelvic dissociation is an extremely rare injury that accounts for ~1% of all spinal fractures and is always the result of high-energy trauma [3]. Due to the high energy of these traumatic events, concomitant injuries are extremely common and there are extremely high rates of neurological deficits and infection as seen in our case report [10,11,13]. The optimal treatment of these injuries has not been well described and usually depends on surgeon preference as well as ability [4,7]. Despite the findings that function, sacral slope, and subjective feelings of pain all improve, patients treated with lumbopelvic fixation still suffer from some level of impairment at follow-up [11].

In our case, we attempted a cantilever technique that resulted in improved sacral slope, patient subjective feelings of pain, and functional improvement. These initial outcomes are consistent with the results of other reduction and fixation attempts [10]. However, shortly after the operation the patient reportedly subjected himself to high biomechanical forces that were likely far too great for his four-rod complex with four points of fixation in his pelvis. This likely resulted in the hardware failure that was revised with a second operation, indicated due to a large hematoma that developed following the patient’s pelvic fracture.

The patient has yet to present with further issues with hardware or traumatic events. However, the absence of complaints to his medical team should not be confused with the absence of severe distress to this patient. In the past 11 years, this patient has undergone eight operations on his spine. Regardless of the potential benefits surgery offers to patients, there are always risks. Reoperation poses significant health risks to patients and results in increased costs [14]. Our team subjected this man to his seventh and eighth spine surgeries, which were related to the fracture and dissociation of his lumbar spine from the pelvis. The consequences of repeated surgery were financial as well as physical.

This case illustrates the need for careful cost-benefit analysis and discussion between spine surgeons and their patients when considering both index spine surgeries as well as revision surgeries. Spinal surgery patients are burdened with heavy costs of care, especially those who undergo operations in acute spinal trauma [15]. Surgeons should not only consider the indications for surgery alongside the complication risks of the specific operation, but they should also consider the financial cost to patients. In hindsight, the correction of this patient's anterior pelvic tilt may better have been treated conservatively or with a fusion in-situ operation that has less of a risk for the complications encountered in this case.

## Conclusions

We present a case of lumbosacral dissociation that was treated with operative fixation using a cantilever technique that resulted in biomechanical failure. The patient’s socioeconomic and living situation likely played a role in the initial presentation as well as the hardware failure after the first operation described here that potentially could have been avoided with adequate assistance from home health, physical, and occupational therapy. This patient stood to benefit from assistance with activities of daily living, including transfers to and from his wheelchair. After revision in a second operation, the patient has not reported any issues with his hardware in over a year postoperatively. Upon reexamination of this case, instead of attempting to correct the chronically developing pelvic tilt, excessive biomechanical forces on the hardware could have been avoided by fusing in-situ.
